# Clinical impact of visceral-to-subcutaneous fat ratio in patients with acute aortic dissection

**DOI:** 10.1371/journal.pone.0226642

**Published:** 2019-12-23

**Authors:** Yusuke Miura, Satoshi Higuchi, Kenichi Matsushita, Toshiya Kariyasu, Haruhiko Machida, Kenichi Yokoyama, Kyoko Soejima, Toru Satoh

**Affiliations:** 1 Department of Cardiology, Kyorin University School of Medicine, Mitaka, Tokyo, Japan; 2 Department of Emergency and General Medicine, Kyorin University School of Medicine, Mitaka, Tokyo, Japan; 3 Department of Radiology, Kyorin University School of Medicine, Mitaka, Tokyo, Japan; International University of Health and Welfare, School of Medicine, JAPAN

## Abstract

**Background:**

Obesity has increased worldwide. Although the visceral-to-subcutaneous fat ratio (VS ratio) is an established risk factor for cardiovascular disease, its clinical impact on the long-term prognosis of patients with acute aortic dissection (AAD) remains unclear.

**Materials and methods:**

This retrospective study included 111 patients with AAD admitted to our hospital from 2013 to 2016. Patients who died during hospitalization, and those diagnosed with Marfan’s syndrome were excluded. Visceral and subcutaneous fat accumulation (VFA, SFA) at umbilical level were calculated on a dedicated workstation. Major Adverse Cardiovascular and Cerebrovascular Events (MACCE) and worsening renal function (WRF) at 3 years were evaluated.

**Results:**

Patient characteristics were as below: age, 73 ± 13; male, 55%; Stanford type A, 53%. Average VFA, SFA, and VS ratio on admission were 98 (52–145) cm^2^, 141 (90–185) cm^2^, and 0.75 (0.47–0.97), respectively. VFA was higher in male than in female (male, 134 [84–179] cm^2^; female, 71 [46–99] cm^2^; *p* < 0.001), whereas SFA was similar (male, 141 [91–174] cm^2^: female, 134 [90–205] cm^2^; *p* = 0.687). VS ratio was also higher in male (male, 0.88 [0.75–1.17]; female, 0.49 [0.39–0.65]; *p* < 0.001). Both MACCE and WRF at 3 years were observed in 17 (15%) and 32 (29%) patients, respectively. Multivariate Cox regression analysis demonstrated that VS ratio tended to be associated with the 3-year MACCE (HR for an increase of 0.5 unit, 1.49; 95% CI, 0.99–2.24; *p* = 0.056). This result persisted in male (HR for an increase of 0.5 unit, 1.54; 95% CI, 0.96–2.48; *p* = 0.073) but not female. The VS ratio was not related to the 3-year WRF.

**Conclusion:**

The VS ratio tends to be associated with the 3-year MACCE in patients with AAD. This finding is inconclusive owing to a small sample and low incidence of adverse events. Further studies with larger samples are needed to confirm the clinical significance of VS ratio.

## Introduction

The increasing prevalence of obesity, which is related to both increased mortality and morbidity [1–3], is a major health problem in both developed and developing countries [4–6]. The severity of obesity is evaluated using various quantitative measures such as body mass index (BMI), abdominal girth, visceral fat accumulation (VFA), and the visceral-to-subcutaneous fat ratio (VS ratio) [7]. Recent studies have reported an adverse impact of visceral fat on cardiovascular and cerebrovascular diseases [8–10], with some studies suggesting that cytokines released from visceral adipocytes may cause inflammation and promote atherosclerosis [11].

Cases of acute aortic dissection (AAD) are relatively rare, with a poor prognosis. The 1 and 10-year survival rates for AAD patients after discharge range from 50% to 80% and 30% to 60%, respectively; the long-term mortality rates are similar for both type A and B dissections [12–15]. Despite the poor prognosis, a majority of patients are only treated with conservative medical therapy to control their blood pressure and heart rates [16]. Patients with abdominal aortic aneurysms have similar VFA and a higher VS ratio compared to those without [17], but few studies have reported a correlation between AAD and these factors. If increased VFA and VS ratios are found to be associated with, or responsible for, subsequent adverse events, these variables could be utilized to predict adverse events or as targets for lifestyle including diet and aerobic exercise [18, 19], pharmacological [20], and surgical [21] interventions. Furthermore, previous studies have shown that there are differences in body fat between men and women [22]. This heterogeneity may provide a different clinical significance of VFA and the VS ratio for male and female. This study aimed to clarify the characteristics including sex differences and the clinical impact of VFA and the VS ratio in patients with AAD.

## Materials and methods

### Study design and population

In this retrospective study, we included data of patients who complained of chest pain with or without back pain due to AAD and were admitted to the Department of Emergency Medicine at the Kyorin University Hospital from January 2013 to December 2016. AAD was diagnosed by at least one cardiologist and one radiologist using contrast-enhanced computed tomography (CT); if CT scans revealed a dissection flap separating a false aortic lumen from the true lumen, the diagnosis was confirmed. If contrast–enhanced CT was not available or was not performed due to contraindications—such as severely impaired kidney function or allergy to the contrast medium—a non-contrast CT scan was performed. On a non-contrast CT scan, findings including the dissection flap and the “calcium sign” [23] were used for confirming the AAD diagnosis. Patients who died during hospitalization or who were diagnosed with Marfan’s syndrome were excluded from the study.

### Data collection

All relevant data of patients was collected, including their characteristics (i.e., age, sex, BMI), significant associated medical conditions (i.e., hypertension, diabetes mellitus, dyslipidemia, atrial fibrillation or flutter, or chronic obstructive pulmonary disease), vital signs, laboratory data, medication history, CT findings (the Stanford classification [24], affected arteries, status of the false lumen [25], and ulcer-like projection (ULP) [26]), oral medications advised at discharge (i.e., calcium channel blockers, beta-blockers, angiotensin-converting enzyme inhibitors, angiotensin II receptor blockers, or other cardiovascular medications), and clinical outcomes (i.e., in-hospital and 3-year mortality, acute myocardial infarction, recurrent aortic dissection, endovascular aortic repair / thoracic endovascular aortic repair, and acute ischemic stroke at 3-year follow-up).

### Follow-up

Follow-up was considered to have begun on the date of discharge. All data in the present study was collected by October 31, 2019. Follow-up was considered complete on the day of the last medical interview, the day of the last examination, or the day on which an endpoint was observed, whichever came first. All data concerning follow-up, all causes of mortality, and adverse clinical events at 3 years was collected from the medical records.

### Evaluation of VFA, SFA, and aortic morphology

Chest, abdominal, and pelvic scans that were performed using the Aquilion^™^ Precision, Aquilion^™^ ONE, Aquilion^™^ Prime, and Aquilion^™^ 64 CT scanners (manufactured by Canon Medical Systems Corporation, Tochigi, Japan) were used for the analysis of aortic morphology on admission and at the 1-year follow-up. The amount of VFA and subcutaneous fat accumulation (SFA) at the umbilical level was also automatically calculated without exposure to any additional radiation, using the dedicated Synapse Vincent version 4.6.0007 (FUJIFILM Medical, Tokyo, Japan) workstation. Each fat area was reviewed and errors such as trace mistakes were corrected by two cardiologists (Y.M. and S.H.) or one radiologist (T.K.), as necessary.

### Definitions

The 3-year Major Adverse Cardiovascular and Cerebrovascular Events (MACCE) was defined as 3-year all-cause mortality, acute myocardial infarction, recurrent aortic dissection, aortic enlargement (for which endovascular aortic repair or thoracic endovascular aortic repair was indicated), or acute ischemic stroke. Worsening renal function (WRF) was defined as an increase in serum creatinine ≥0.3 mg/dL from baseline at discharge [27, 28]. Dyslipidemia was diagnosed if the low-density lipoprotein (LDL) cholesterol level (calculated as total cholesterol − high-density lipoprotein (HDL) cholesterol − triglyceride/5) was ≥140 mg/dL, the HDL cholesterol level was <40 mg/dL, or the triglyceride level was ≥150 mg/dL. Hypertension was defined as systolic blood pressure (SBP) ≥140 mmHg and a diastolic blood pressure ≥90 mmHg [29]. Patients were considered diabetic if they reported diagnosis of diabetes mellitus (DM) or use of anti-diabetic drugs on the questionnaire. Patients with fasting plasma glucose level ≥126 mg/dL or with glycosylated hemoglobin (HbA1c) level ≥6.5% were also included in this subset [30].

### Endpoints

The primary endpoint was the MACCE at 3 years, whereas the secondary endpoint was the WRF at 3 years.

### Ethical principles

The study protocol conforms to the ethical guidelines of the 1975 Declaration of Helsinki and is in line with the Ethical Guidelines for Epidemiological Research prescribed by the Japanese government. The study was approved by the ethics committee at Kyorin University. According to the guidelines, the study satisfied the conditions needed to waive the requirement of written informed consent from individual participants. The waiver was approved by the ethics committee.

### Statistical analysis

Numerical data which followed a normal distribution are reported as mean ± standard deviation, whereas non-normally distributed data variables are reported as median and interquartile ranges (Q1–Q3). Categorical variables are expressed as absolute numbers (percentages). Continuous variables were analyzed using the unpaired Student’s *t* test or Wilcoxon signed-rank test, as appropriate. Fisher’s exact test and the chi-squared (χ^2^) test were used to analyze categorical variables. Associations of BMI with VFA, SFA, and VS ratio were assessed using Spearman’s rank correlation coefficient. The correlation between primary endpoint and VS ratio was assessed using uni- and multivariate Cox regression analyses and expressed as a hazard ratio (HR) with a 95% confidence interval (CI). All clinical variables were subjected to multivariate Cox regression analysis with least absolute shrinkage and selection operator (LASSO). Statistical significance was set at *p* < 0.05. All statistical analyses were carried out using Stata^®^ software, version 14 (StataCorp LLC, College Station, TX) and R version 3.6.1 (R Foundation for Statistical Computing, Vienna, Austria).

## Results

### Patients’ characteristics

Of the 125 patients diagnosed with AAD, 14 died during hospitalization. Therefore, the study included total 111 individuals (mean age, 73 ± 13; male, 55%; Stanford type A, 53%). The patients’ characteristics are recorded in Table 1. The mean BMI was 23 ± 4 kg/m^2^. The median VFA and SFA on admission were 98 (52–145) cm^2^ and 141 (90–185) cm^2^, respectively. The median VS ratio of the study group was 0.75 (0.47–0.97).

**Table 1:** Patient characteristics.

	All	Male	Female	p value
	(n = 111)	(n = 61)	(n = 50)	
Age, years	73 ± 13	69 ± 13	77 ± 12	0.001
Male, n (%)	61 (55)	61 (100)	0 (0)	< 0.001
Systolic blood pressure, mmHg	144 ± 42	150 ± 40	137 ± 44	0.041
Diastolic blood pressure, mmHg	80 ± 24	84 ± 25	74 ± 22	0.042
Heart rate, beats per minute	75 ± 20	78 ± 21	71 ± 18	0.225
Hypertension, n (%)	78 (70)	41 (67)	37 (74)	0.436
Diabetes mellitus, n (%)	13 (12)	7 (11)	6 (12)	1.000
Dyslipidemia, n (%)	27 (24)	15 (25)	12 (24)	1.000
Smoker, n (%)	51 (46)	40 (66)	11 (22)	< 0.001
Body mass index, kg/m^2^	23 ± 4	24 ± 3	22 ± 4	0.003
Findings of computed tomography				
Stanford A, n (%)	59 (53)	28 (46)	31 (62)	0.091
Involved renal arteries, n (%)	65 (59)	40 (66)	25 (50)	0.097
Involved superior/inferior mesenteric arteries, n (%)	78 (70)	45 (74)	33 (66)	0.373
Visceral fat accumulation, cm^2^	98 (52–145)	134 (84–179)	71 (46–99)	< 0.001
Subcutaneous fat accumulation, cm^2^	141 (90–185)	141 (91–174)	134 (90–205)	0.687
Visceral-to-subcutaneous fat ratio	0.75 (0.47–0.97)	0.88 (0.75–1.17)	0.49 (0.39–0.65)	< 0.001
Laboratory data				
High-density lipoprotein, mmol/L	1.19 (1.01–1.50)	1.14 (1.01–1.34)	1.22 (1.01–1.60)	0.244
Low-density lipoprotein, mmol/L	2.72 (2.33–3.31)	2.72 (2.17–3.44)	2.69 (2.40–3.10)	0.822
Hemoglobin A1c, %	5.8 ± 0.6	5.7 ± 0.5	5.9 ± 0.7	0.184
Albumin, g/L	38 ± 5	39 ± 4	38 ± 5	0.077
Uric acid, μmol/L	318 ± 93	325 ± 89	310 ± 99	0.333
C-reactive protein (baseline), mg/L	1.6 (0.7–4.6)	1.7 (0.8–4.0)	1.3 (0.6–6.3)	0.799
C-reactive protein (peak), mg/L	146.9 (67.0–209.0)	157.1 (73.3–231.2)	128.3 (53.9–193.1)	0.092
D-dimer, μg/L	4510 (1570–15050)	3650 (1500–15050)	5305 (1610–19090)	0.524
Hemoglobin at discharge, g/L	113 ± 17	110 ± 16	116 ± 17	0.159
Serum creatinine at discharge, μmol/L	74 (58–94)	78 (67–108)	60 (54–80)	< 0.001
Medication at discharge				
Calcium channel blocker, n (%)	81 (73)	49 (80)	32 (64)	0.085
β blocker, n (%)	92 (83)	54 (89)	38 (76)	0.127
Renin-angiotensin system inhibitors, n (%)	52 (47)	33 (54)	19 (38)	0.126
α channel blocker, n (%)	14 (13)	9 (15)	5 (10)	0.570
Furosemide, n (%)	32 (29)	17 (28)	15 (30)	0.836
Antiplatelet therapy, n (%)	12 (11)	6 (10)	6 (12)	0.766

### Sex-related differences in VS ratio

VFA was higher in male (male, 134 [84–179] cm^2^; female, 71 [46–99] cm^2^; *p* < 0.001), while SFA was similar (male, 141 [91–174] cm^2^: female, 134 [90–205] cm^2^; *p* = 0.687). VS ratio was also higher in male (male, 0.88 [0.75–1.17]; female, 0.49 [0.39–0.65]; *p* < 0.001).

### Correlation of BMI with VFA, SFA and VS ratio

**Fig 1:**
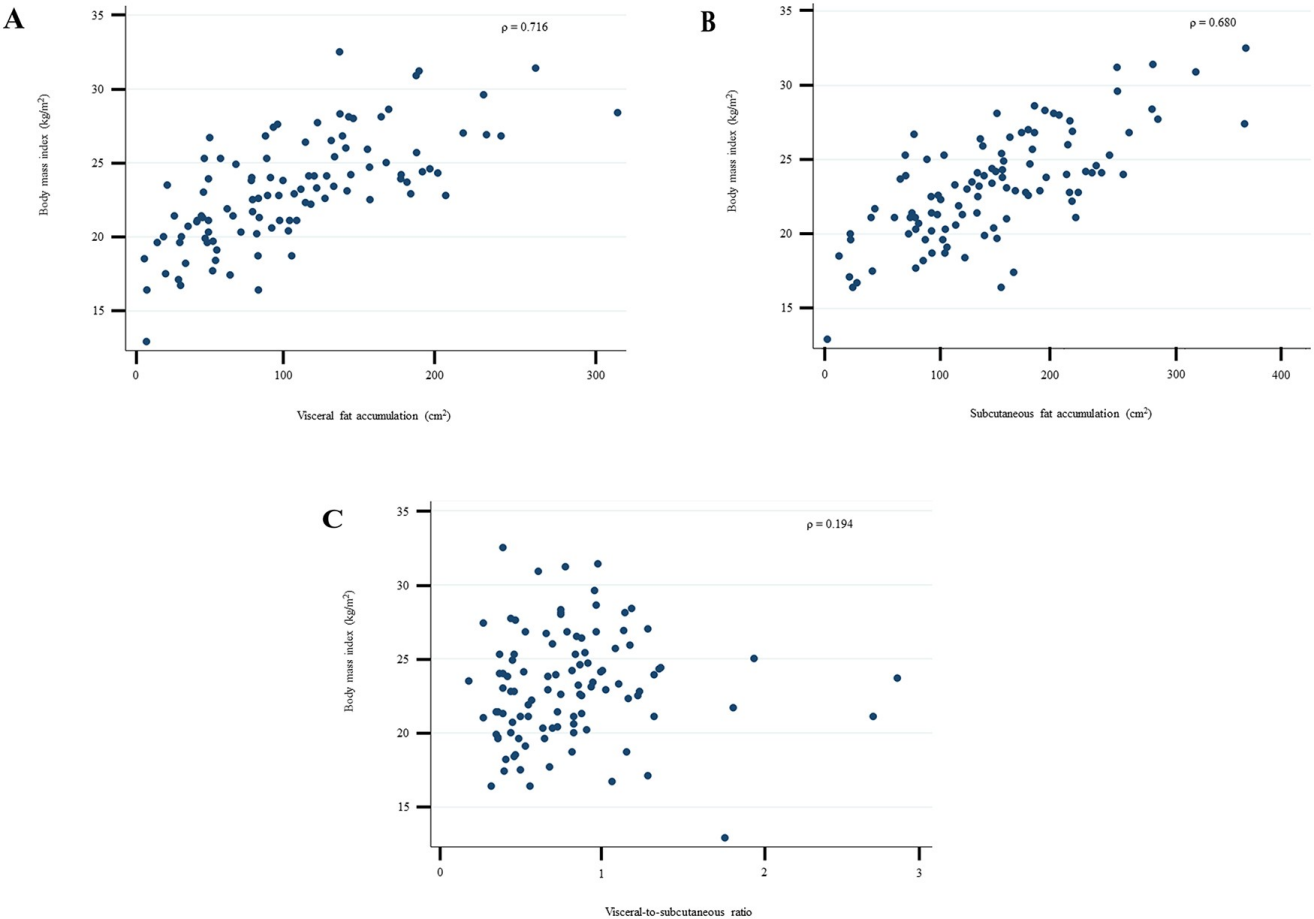
An association between body mass index and each of the fat indices. Body mass index correlated with (a) visceral fat accumulation and (b) subcutaneous fat accumulation, but did not correlate with (c) visceral-to-subcutaneous fat ratio.

BMI was correlated with both VFA and SFA (ρ = 0.716 and ρ = 0.680, respectively), while only a weak association was observed with the VS ratio (ρ = 0.194) (Fig 1a–1c).

### Clinical impact of VS ratio on 3-year MACCE and WRF

The primary and secondary endpoints were observed in 17 (15%) and 32 (29%) patients, respectively. The median follow-up period was 1037 (278–1321) days. The univariate Cox regression analysis of 3-year MACCE and WRF are shown in Tables 2 and 3. While VS ratio tended to be associated with the primary endpoint (HR for an increase of 0.5 unit, 1.52; 95% CI, 0.99–2.33; *p* = 0.055) and the secondary endpoint (HR for an increase of 0.5 unit, 1.39; 95% CI, 0.99–1.96; *p* = 0.058), VFA and SFA were not related to such endpoints. Multivariate Cox regression analysis for 3-year MACCE demonstrated similar results (HR for an increase of 0.5 unit, 1.49; 95% CI, 0.99–2.24; *p* = 0.056). This result persisted in male (HR for an increase of 0.5 unit, 1.54; 95% CI, 0.96–2.48; *p* = 0.073), but not in female (HR for an increase of 0.5 unit, 0.67; 95% CI, 0.10–4.43; *p* = 0.677). The VS ratio was not related to the 3-year WRF after adjustment.

**Table 2:** Cox regression analysis for 3-year MACCE.

	Univariate			Multivariate		
	HR	95% CI	p value	HR	95% CI	p value
Age	1.06	1.01–1.12	0.029	1.06	1.01–1.13	0.028
Male	1.89	0.65–5.54	0.244	NA		
Body Mass Index (an increase of 1 kg/m^2^)	1.01	0.88–1.17	0.849	NA		
Hypertension	1.47	0.41–5.29	0.552	NA		
Diabetes Mellitus	2.27	0.73–7.11	0.158	NA		
Dyslipidemia	2.15	0.77–5.96	0.143	NA		
Atrial fibrillation	1.60	0.51–5.04	0.425	NA		
**Computed tomography**						
Stanford A	0.73	0.27–1.99	0.538	NA		
Visceral fat accumulation (an increase of 10 cm^2^)	1.02	0.95–1.10	0.543	NA		
Subcutaneous fat accumulation (an increase of 10 cm^2^)	0.96	0.89–1.04	0.291	NA		
Visceral-to-subcutaneous fat ratio (an increase of 0.5 units)	1.52	0.99–2.33	0.055	1.49	0.99–2.24	0.056
Visceral fat accumulation ≥100 cm^2^	1.65	0.59–4.57	0.337	NA		
Ulcerlike projection	2.46	0.83–7.26	0.104	NA		
Renal arteries involvement	1.20	0.43–3.33	0.722	NA		
Superior/inferior mesentric arteries involvement	1.34	0.43–4.21	0.614	NA		
**Laboratory data**						
Peak C-reactive protein level, 10 mg/L	0.99	0.94–1.05	0.737	NA		
Serum creatinine level at discharge, an increase of 10 μmol/L	1.02	0.98–1.07	0.313	NA		
Hemoglobin level at discharge, an increase of 10 g/L	0.91	0.67–1.23	0.547	NA		
Albumin level, an increase of 10 g/L	1.61	0.74–3.52	0.231	NA		
**Medication at discharge**						
Calcium channle blocker	0.71	0.25–2.05	0.529	NA		
Beta blocker	0.42	0.12–1.53	0.188	NA		
Renin-angiotensin system inhibitors	1.05	0.37–3.01	0.929	NA		
Alpha blocker	0.47	0.06–3.60	0.467	NA		
Furosemide	1.26	0.45–3.51	0.654	NA		
Antiplatelet therapy	1.87	0.41–8.46	0.418	NA		

CI, confidence interval; HR, hazard ratio; MACCE, major adverse cardiovascular and cerebrovascular events; NA, not applicable

Multivariate Cox regression analysis was adjusted for age and visceral-to-subcutaneous fat ratio.

**Table 3:** Cox regression analysis for 3-year WRF.

	Univariate			Multivariate		
	HR	95% CI	p value	HR	95% CI	p value
Age	1.03	0.99–1.06	0.110	NA		
Male	1.05	0.51–2.16	0.903	NA		
Body Mass Index (an increase of 1 kg/m^2^)	1.01	0.91–1.13	0.812	NA		
Hypertension	1.31	0.56–3.06	0.539	NA		
Diabetes Mellitus	1.60	0.61–4.20	0.339	NA		
Dyslipidemia	0.44	0.15–1.28	0.130	NA		
Atrial fibrillation	0.98	0.40–2.41	0.969	NA		
**Computed tomography**						
Stanford A	0.92	0.45–1.88	0.824	NA		
Visceral fat accumulation (an increase of 10 cm^2^)	1.00	0.94–1.06	0.938	NA		
Subcutaneous fat accumulation (an increase of 10 cm^2^)	0.96	0.91–1.02	0.187	NA		
Visceral-to-subcutaneous fat ratio (an increase of 0.5 units)	1.39	0.99–1.96	0.058	NA		
Visceral fat accumulation ≥100 cm^2^	1.38	0.67–2.82	0.378	NA		
Ulcerlike projection	1.37	0.55–3.40	0.496	NA		
Renal arteries involvement	1.01	0.49–2.07	0.983	NA		
Superior/inferior mesentric arteries involvement	1.07	0.49–2.34	0.867	NA		
**Laboratory data**						
Peak C-reactive protein level, 10 mg/L	0.99	0.95–1.03	0.485	NA		
Serum creatinine level at discharge, an increase of 10 μmol/L	1.03	1.00–1.07	0.072	1.04	1.01–1.08	0.024
Hemoglobin level at discharge, an increase of 10 g/L	0.97	0.77–1.22	0.812	NA		
Albumin level, an increase of 10 g/L	1.17	0.64–2.13	0.613	NA		
**Medication at discharge**						
Calcium channle blocker	0.45	0.22–0.92	0.028	NA		
Beta blocker	0.55	0.21–1.45	0.227	NA		
Renin-angiotensin system inhibitors	0.75	0.35–1.57	0.440	NA		
Alpha blocker	1.77	0.72–4.37	0.217	NA		
Furosemide	2.27	1.12–4.63	0.024	2.16	1.01–4.63	0.048
Antiplatelet therapy	2.03	0.70–5.91	0.196	NA		

CI, confidence interval; HR, hazard ratio; WRF, worsening renal function; NA, not applicable

Multivariate Cox regression analysis was adjusted for serum creatinine level and prescription of furosemide at discharge. Visceral-to-subcutaneous fat ratio was omitted by LASSO.

### Comparison between VS ratios recorded on admission and 1 year from discharge

**Fig 2:**
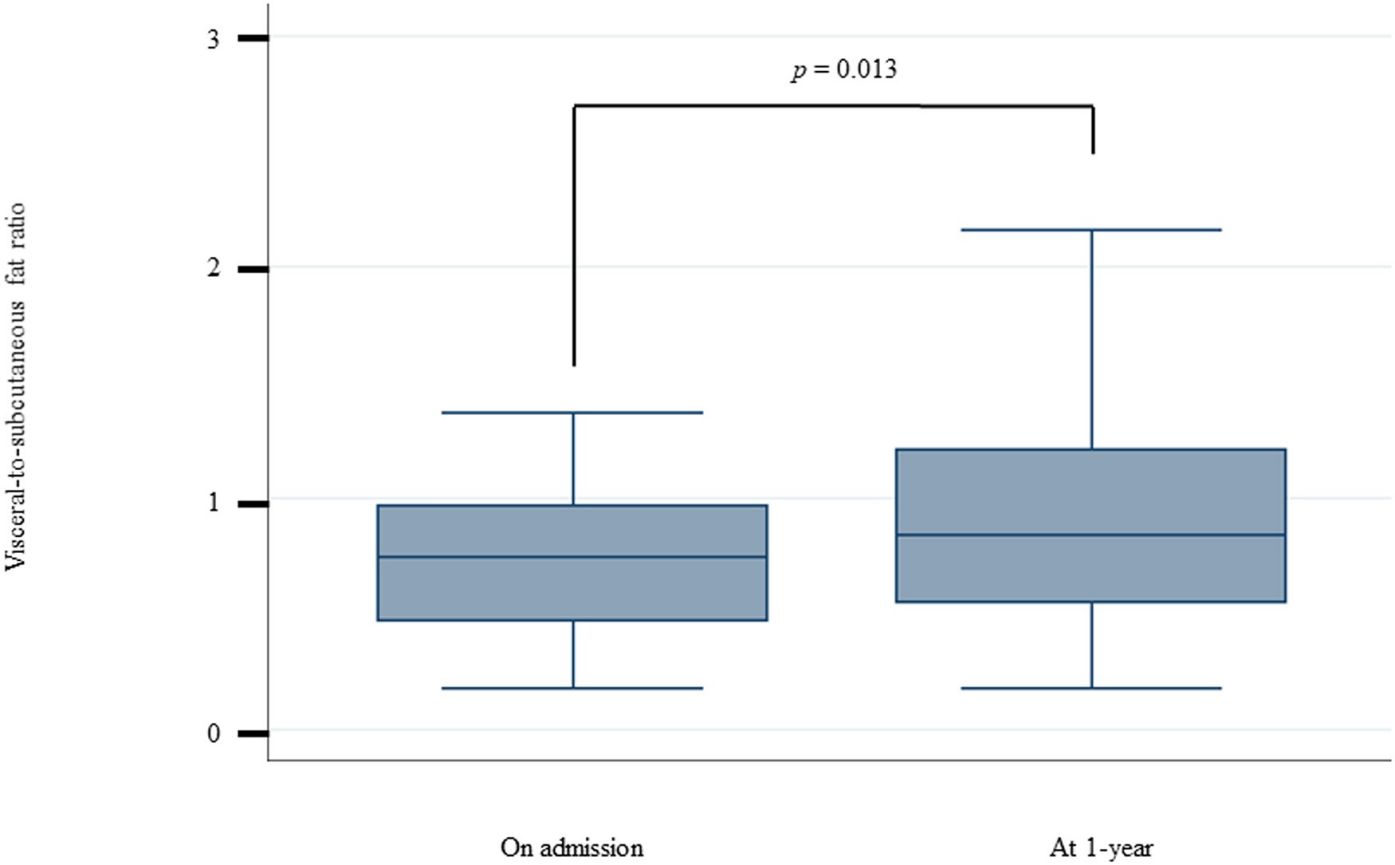
The change in visceral-to-subcutaneous fat ratio with time. The visceral-to-subcutaneous fat ratio level at 1-year was significantly higher than that recorded at baseline.

The median VS ratios were estimated to be 0.79 (0.49–1.03) on admission and 0.84 (0.55–1.21) 1 year after discharge (*p* = 0.013) (Fig 2).

## Discussion

This study suggests the potential clinical impact of VS ratio on the 3-year MACCE in patients with acute aortic dissection. Notably, it is easy to evaluate this index without any additional radiation exposure or cost, with the main purpose of CT imaging being a confirmation of the diagnosis of AAD.

### Prognostic factors related to 3-year MACCE: Significance of VS ratio

The present study demonstrated that VS ratio tended to be associated with the 3-year MACCE in patients with AAD; on the other hand, VFA was not related to the 3-year MACCE. Notably, VS ratio was weakly associated with BMI. It is well known that BMI is rather heterogeneous and that individuals with similar BMI may have different atherosclerotic risks and morbidities [31]. Furthermore, no significant association between VFA and 3-year MACCE was observed. This may be due to type II errors introduced both by small sample size and clinical endpoint, which lowered the statistical power of the research. Additionally, the different roles of VFA and SFA might have affected the results. In fact, VFA is generally more prevalent in individuals with obesity than SFA; however, there is a healthy obese phenotype characterized by a greater volume of SFA. SFA secretes hormones such as leptin, which exerts some beneficial metabolic effects [32, 33]. Previous studies have demonstrated that SFA may also play a role in absorbing or neutralizing inflammatory products generated in the visceral fat [34, 35]. Therefore, the VS ratio can be considered to be an indicator of the interaction between VFA and SFA, and may be a more useful marker for the 3-year MACCE compared with VFA. Interestingly, VS ratio increased during follow-up in this study; although the clinical significance of the results remains unclear, patients with higher VS ratios may experience subsequent clinical adverse events [3].

According to previous studies [36–38], conflicting results regarding an association of age with long-term mortality in those who underwent AAD have been reported. However, aging is known to be an important risk factor for atherosclerosis [39]; it would be plausible that aging was associated with the 3-year MACCE in our study.

### Prognostic factors related to the 3-year WRF

The present study showed that serum creatinine level and prescription of furosemide at discharge were related to 3-year WRF, while VS ratio was not. A previous study indicated that WRF was related to subsequent mortality, as well as that higher baseline glomerular filtration rate was associated with more frequent WRF [40]. Loop diuretics were also an independent predictor of WRF [41, 42]. Furthermore, mortality increased according to the dose of loop diuretic [43]. Diuretic therapy may be associated with a reduction in the effective circulating arterial volume with little effect on central venous pressure, resulting in a net reduction of renal perfusion pressure and WRF. Patients with renal impairment at baseline might be influenced by such reduced renal perfusion more than those without the complication.

### Sex-related differences in VS ratio

The present study indicated sex-related differences in VS ratios among individuals with AAD. Previous studies have indicated that male in the general population have more VFA than female [44, 45]. Men are more likely to accumulate adipose tissue in the abdomen, whereas women usually accumulate adipose tissue in the gluteal and femoral regions.[46] Interestingly, de Santana et al. [47] demonstrated a different clinical impact of VFA between the two sexes. In that same study, VS ratio tended to be associated with increased 3-year MACCE in male but not in female. The sex-related differences in VS ratio may be partially explained by sex-specific endocrinological differences. This speculation can be supported by evidence from transsexuals treated with sex hormones. Female-to-male transsexuals who have been treated with testosterone showed a progressive shift in body fat distribution from the female to the male pattern [48, 49].

### Limitations

This was a retrospective study conducted at a single center. Therefore, some biases may have been introduced. First, the small sample size with the unmatched design is a limitation of the present study, which may have caused a bias in identifying factors indicating prognosis other than VS ratio, impaired kidney function, and prescription of beta-blockers. Moreover, the incidence of 3-year MACCE was rather low. Large numbers of censored values reduce the power of survival analysis. There could be factors potentially confounding the association between VS ratio and clinical adverse events which we were not able to identify and to take into account due to the number of 3-year MACCE, rather small for performing multivariate analyses. However, LASSO might reduce the impact of selection bias in multivariate analysis. Secondly, both Stanford types A and B of aortic dissection were assessed simultaneously. However, previous studies reported that the sub-types may have shown similar long-term mortality rates [12, 15, 50–52]. Thirdly, the present study was conducted at a single clinical setting. Thus, its results cannot be generalized to the population at large. Finally, the present study could not provide a cut-off value of the VS ratio as a significant predictor. Although the results of this small study can be considered preliminary and further studies with a larger sample size would be necessary in order to confirm our results, they would suggest the importance of VS ratio in AAD.

## Conclusion

The VS ratio tends to be associated with the 3-year MACCE in patients with AAD. This finding is not conclusive, owing to a small number of patients included in the study as well as to the low incidence of clinical adverse events. Further studies with greater sample sizes are needed in order to confirm the clinical significance of VS ratio and its different impact based on sex.
